# Summary and Analysis of Relevant Evidence for Nondrug Nursing Programs in Neonatal Operational Pain Management

**DOI:** 10.1155/2022/7074500

**Published:** 2022-05-27

**Authors:** Zhuo Yang, Yinan Fu, Yueqi Wang

**Affiliations:** ^1^Emergency Intensive Care Unit, The First Hospital of Jilin University, Changchun, Jilin 130000, China; ^2^Neonatal Intensive Care Unit, The First Hospital of Jilin University, Changchun, Jilin 130000, China

## Abstract

**Purpose:**

To summarize the relevant evidence for nondrug nursing programs in neonatal operational pain management.

**Methods:**

Computer search for the literature on neonatal procedural pain from 2015 to 2020 in Up To Date, JBI, NICE, SIGN, RNAO, NGC, PubMed, Cochrane Library, CNKI, and Wanfang database was conducted. All literature works that may meet the inclusion criteria were independently evaluated by two researchers to determine the quality grade of the articles.

**Results:**

Finally, 9 literature works were extracted, including 4 guidelines, 3 systematic reviews, and 2 evidence summaries. The relevant contents of the literature were extracted and summarized, and 20 pieces of the best evidence were obtained.

**Conclusion:**

Breast feeding, sweetener, Kangaroo mother care, sensory stimulation, nonnutritive sucking, and other nondrug nursing programs can reduce the neonatal operational pain, which has guiding significance in neonatal operational pain management.

## 1. Introduction

Since the development of the neonatal pain conduction pathway is not perfect and the inhibition of pain is also lacking, the neonate often produces relatively strong pain response [1]. The pain tolerance of a neonate is lower than that of children of other ages, and the pain threshold level is 50% –70% of that of adults [2]. Studies have shown that compared with adults, the pain perceived by neonates is more severe and lasting [3]. After birth, neonates will experience operational pain in different ways, such as hepatitis B vaccine injection, vitamin injection, fundus screening, and heel blood collection. In addition, NICU children will also face other painful operations such as tracheal intubation, central venous puncture, and lumbar puncture [4, 5]. Repeated painful operations are associated with programmed changes in the hypothalamic-pituitary-adrenal axis (HPA). Cruz's team conducted an epidemiological survey of NICU neonates, and the results showed that the operational pain was 7.5–17.3 times per day for each neonate within 14 days before birth [6]. Pain may cause unstable physiological outcomes in the newborn, such as increased heart rate, shortness of breath, changes in intracranial blood volume, and abnormal changes in blood pressure. Repeated painful operations will lead to stress reaction of neonates, and severe cases will be detrimental to the development of neonates. After experiencing long-term operational pain, neonates may suffer from sleep disorders, difficulty in feeding, loss of appetite, and other problems, and at the same time, they may have long-term effects such as changes in nerve development, decreased cognitive ability of behavior, decreased perception ability, depression, and anxiety [7, 8]. In recent years, more attention has been paid to the pain of neonates. Although the medical staff's awareness of pain has been improved, nurses seldom take measures to relieve the pain of neonates in the actual treatment and nursing process. Roofthooft's team found that only about 36.6% of patients will be treated with analgesia for operational pain in clinic [9]. For neonatal operational pain, a nondrug nursing program is a safe and effective intervention measure, which can appropriately reduce the use of drug intervention and reduce the harm of drugs to neonates. The purpose of this study is to summarize the relevant evidence for nondrug nursing programs in neonatal operational pain management, so as to provide reference for guiding the management of neonatal operational pain.

## 2. Materials and Methods

### 2.1. Identify Evidence-Based Problems

According to PIPOST tools, clinical problems are transformed into evidence-based problems: ① P (population): neonates whose birth age is ≤28 days; ② I (intervention): intervention measures to relieve pain; ③ P (professional): pediatric nurses, physicians, and family members of neonates; ④ O (outcome): O_1_ (heart rate, pulse, oxygen saturation, etc.) and O_2_ (duration of painful face, duration of crying, etc.); ⑤ S (setting): pediatric ward; ⑥T (type of evidence): guideline, systematic reviews, and evidence summary.

### 2.2. Evidence Retrieval Strategy

With “procedural pain, pain, analgesia, non-drug analgesia, neonate, neonatology department” as the English keywords, the retrieved databases include the following: Up To Date, JBI Evidence-Based Health Care Center database, National Institute for Clinical Excellence (NICE), Scottish Interhospital Guide Network (SIGN), Registered Nurses' Association of Ontario (RNAO), National Guideline Clearinghouse (NGC), PubMed, Cochrane Library, CNKI, and Wanfang database. The retrieval period was from 2015 to 2020.

### 2.3. Literature Inclusion Criteria and Exclusion Criteria

Inclusion criteria are as follows: the research object includes clinical guidelines, evidence summary, and systematic reviews of neonates with operational pain. Exclusion criteria are as follows: Studies that involve nondrug nursing programs but do not indicate specific interventions, summary, abstract, draft, interpretation of evidence, research proposal, research with no clear conclusion, and duplicate evidence.

### 2.4. Literature Quality Evaluation Standard

① Appraisal of Guidelines Research and Evaluation in Europe II (AGREE II) was used to evaluate the clinical guideline [10]. The evaluation tool includes 6 areas: scope and purpose, participants, rigor of formulation, clarity, applicability, and editorial independence. It includes 23 items, with a score of 1 to 7 points. The higher the score, the higher the degree of agreement. The standardized score of each field was calculated. The recommendation grades are divided into Grade A (directly recommended), Grade B (recommended after modification), and Grade C (not recommended).

② Assessment of multiple systematic reviews (AMSTAR) was used to evaluate the systematic reviews [11]. It includes 11 items. The items are scored by yes: 1 points; unclear or not mentioned: 0.5 points; and no: 0 points. The highest score is 11 points. The overall quality evaluation is divided into high quality, medium quality, and low quality. 0–4 points are low quality, 5–8 points are medium quality, and 9–11 points are high quality.

③ All the literature potentially meeting the inclusion criteria was independently evaluated by 2 researchers trained through a national evidence-based nursing curriculum to determine the quality grade of the articles. For literature works that are difficult to reach consensus, the third researcher trained through a national evidence-based nursing curriculum will participate in the discussion to reach consensus. The inclusion principle of literature evidence included the following: evidence-based evidence, high-quality evidence, and newly published evidence should be given priority.

④ The JBI evidence recommendation level system was used for evidence summary [12]. The level of evidence is divided into 1–5 levels. The higher the level, the lower the grade of evidence. The FAME structure based on JBI determines the division of evidence: Grade A (strong recommendation) and Grade B (weak recommendation).

## 3. Results

### 3.1. Retrieval of Results

A total of 402 literature works were obtained in the initial examination. After reading the title, abstract, and full text, the unqualified literature works were eliminated. Finally, 9 literature works were extracted, including 4 guidelines, 3 systematic reviews, and 2 evidence summaries. The basic characteristics of the included literature works are shown in Table 1.

### 3.2. Quality Evaluation of Guidelines

The total quality scores of the 4 guidelines are ≥5 points, so it is recommended to use these 4 guidelines as shown in Table 2.

### 3.3. Quality Evaluation of Systematic Reviews

The problems of the 3 systematic reviews are as follows: the grey literature is not considered in the inclusion criteria, and the possibility of publication bias has not been fully assessed. The 3 systematic reviews are all of high quality, can clearly put forward relevant evidence-based questions, and are scientific and authentic as shown in Table 3.

### 3.4. Evidence Description and Summary

In the process of evidence summary, the 2 evidence summaries included in this study both adopt the original evidence level and recommendation level. The relevant contents of the literature were extracted and summarized, and 20 pieces of the best evidence were obtained. The summary of evidence is shown in Table 4.

## 4. Discussion

### 4.1. Breast Feeding

Breast feeding can be divided into direct breast feeding and indirect breast feeding. The method can promote the mother-infant contact, it can provide physical and psychological comfort for neonates, and it does not increase any medical cost. Ponce-Garcia's team believed that breast feeding can reduce the probability of sleep and breathing disorder in children and significantly shorten the duration of crying in children [22]. Peng's team found that compared with NNS, breast feeding had a better analgesic effect after heel-sticking operation for premature infants [23]. Evidence 4 shows that the sweetener of breast feeding is lactose secreted by the mother. Although lactose does not have the analgesic effect of glucose and sucrose, breast feeding can relieve pain through taste, skin contact, and other ways.

### 4.2. Sweetener

Sweetener has become a commonly used analgesic measure for neonates, and its possible mechanism is that it stimulates an oral sense of taste, stimulates oral tactile receptors, and triggers the release of endogenous opioids. The opioid receptor is a part of the endorphin system, and activating the receptor can reduce the pain sensation of the HPA axis of neonates [24]. Generally speaking, sweeteners relieve pain through the taste at the tip of the tongue. Therefore, taking sweeteners through the gastric tube has no analgesic effect, and they need to be taken orally to relieve pain [25]. Uzelli's team studied premature infants who needed intramuscular injection and found that oral glucose had a positive effect on reducing the pain score, reducing crying time, and improving physiological indicators. At the same time, they believe that giving premature infants the lowest dose of glucose can also relieve pain to some extent [26]. Vezyroglou's team believed that although the sweetener has a good analgesic effect, no serious adverse events have occurred. However, the long-term effects of sweeteners on development and neurological function are still unknown, and the dosage, concentration, and the use time of sweeteners require attention [27].

### 4.3. KMC

KMC refers to the nursing method of placing the newborn's whole body naked on the mother's chest for maximum skin-to-skin contact [28]. KMC relies on various forms of stimulation, such as tactile sensation, warm sensation, and hearing, to activate the neurochemical system, so as to maintain the body temperature of neonates and provide neonates with sufficient warmth and security [29]. At the same time, this nursing method can effectively control the programmed change of the HPA axis by regulating the pressure-regulating system involved in the painful experience, thus effectively blocking the pain sensation of neonates [30]. According to the research of Montealegre-Pomar's team, compared to usual care, KMC can reduce the mortality of premature infants, prevent apnea, and reduce the operational pain score [31]. Pandita's team implemented KMC when the baby was vaccinated and found that the crying time of the baby was obviously reduced and the pain score was reduced [32]. Taddio's team observed 736 neonates, and they finally came to the conclusion that KMC is beneficial to relieve acute pain during operation and KMC can be used when neonates are vaccinated [33]. In addition, clinically, some mothers are in a high-risk state, so they cannot perform KMC on neonates in person. In this case, fathers or other postpartum mothers can perform KMC on the newborns. Evidence 14 proves that there is no difference in the analgesic effect between alternate KMC and KMC. Research by Murmu's team also confirmed this conclusion [34]. It is worth noting that evidence 13 holds that KMC should be implemented as soon as possible and as long as possible for low-birth-weight infants. However, this study is an expert consensus, the level of evidence is low, and more research is needed to confirm it. In clinical practice, the use time of KMC should be determined according to the specific conditions of neonates, disease characteristics, clinical environment, and other factors.

### 4.4. Sensory Stimulation

Sensory stimulation is the primary way of nondrug intervention for neonatal pain. Individualized dependence on sensory intervention can control the programmed change of the HPA axis to a certain extent. Sensory stimulation mainly include touch (stroking the face and back of neonates), taste (giving the neonatal breast milk or sweetener), hearing (talking to the neonates and playing the mother's heartbeat recording), and vision (looking at the neonates) [35]. Qiu's team found that after the intervention of touch and music for premature infants, the pain of premature infants was alleviated, and the main mechanism was that the concentration of *β*-endorphin in the pituitary gland increased significantly [36]. Massage for neonates can reduce the levels of cortisol and norepinephrine in the serum to relieve pain and promote the physique and growth and development of neonates; especially, the upper limb massage has a better analgesic effect [37]. van der Heijden's team proposed that music therapy can stimulate the auditory receptors of premature infants and transmit music stimulation to the pituitary gland, thereby promoting the release of endorphins, catecholamines, and other substances from the pituitary gland, thus alleviating the pain [38]. Kurdahi's team believed that playing music that mothers hear during pregnancy for neonates can relieve pain [39]. Evidence 18 of this study also reflects this view, but the analgesic effect of this method is still unclear. In the implementation of music therapy, the selected music should be comfortable and slow, the melody should be harmonious, and stimulating and loud music should be avoided. White noise is a relatively new method to relieve pain, which can simulate the sound environment in the mother's body and make the neonates have the feeling in the mother's womb again, which has a positive effect on calming the mood of the neonates [40].

### 4.5. NNS

NNS is a method of increasing the sucking action of a neonate by gently placing a pacifier into the mouth of neonates. During the NNS intervention, no breast milk or formula was inhaled [41]. Carbajal's team research showed that NNS has a good analgesic effect, which may be due to the increase in endogenous endorphins [42]. Gao's team pointed out that NNS stimulated the release of 5-HT in the mouth, which relieved some pain, but when the neonate was in severe pain, it often opened its mouth to cry and could not suck. In this case, the appeasement of NNS was ineffective [43]. Pillai's team believed that the quality of evidence for analgesia of NNS in premature infants is low, and the analgesic effect needs to be further explored [44].

## 5. Conclusion

To sum up, breast feeding, sweetener, KMC, sensory stimulation, NNS, and other nondrug nursing programs can reduce the operational pain of neonates, which is characterized by low-risk, simple, and easy implementation. Most of the evidence in this study is Grade A recommendation, which has guiding significance in neonatal operational pain management. In clinical work, it is necessary to increase the attention of nurses to neonatal procedural pain and to adopt a variety of methods to implement comprehensive pain nursing interventions for neonates. In addition, in the process of using evidence, medical staff should fully consider the own situation of neonates, department environment, and other factors, and they should use high-quality evidence-based evidence to improve clinical practice.

## Figures and Tables

**Table 1:** Basic characteristics of the included literature works.

Included literature	Research contents	Source of evidence	Nature of evidence	Year of publication
Taddio et al. [13]	Reduce pain during vaccination	NGC	Guideline	2015
Lago et al. [14]	Nondrug analgesic intervention of common acupuncture in neonates	PubMed	Guideline	2017
Lim et al. [15]	Prevention and management of neonatal operational pain	PubMed	Guideline	2017
ENA Clinical Practice Guideline Committee et al. [16]	Acupuncture or slight manipulation pain intervention for pediatric patients	PubMed	Guideline	2019
Harrison et al. [17]	A sweetener is used for acupuncture pain of children aged 0–16 years	Cochrane Library	Systematic review	2015
Stevens et al. [18]	Application of sucrose in neonatal operational pain	Cochrane Library	Systematic review	2016
Johnston et al. [19]	Kangaroo mother care (KMC) for neonatal operational pain	Cochrane Library	Systematic review	2017
JBI [20]	Breast feeding can relieve neonatal operational pain	JBI	Evidence summary	2019
JBI [21]	KMC is used for low birth weight infants	JBI	Evidence summary	2020

**Table 2:** Quality evaluation of the guideline.

Included literature	Total quality score	Recommended grade
Taddio et al. [13]	6 points	Grade A
Lago et al. [14]	5.5 points	Grade A
Lim et al. [15]	5.5 points	Grade A
ENA Clinical Practice Guideline Committee et al. [16]	5 points	Grade B

**Table 3:** Quality evaluation of systematic reviews.

AMSTAR	Harrison et al. [17]	Stevens et al. [18]	Johnston et al. [19]
Whether to formulate the preliminary design plan?	1 point	1 point	1 point
Whether the research selection and data selection are repeatable?	1 point	1 point	1 point
Whether to carry out a comprehensive retrieval strategy?	1 point	1 point	1 point
Whether the grey literature is considered in the inclusion criteria?	0 points	0 points	0 points
Whether to describe the characteristics of the included study?	1 point	1 point	1 point
Whether to provide a list of the included and excluded literature?	1 point	1 point	1 point
Whether to evaluate the scientificity of the included study?	1 point	1 point	1 point
Whether the scientificity of the included study was properly applied in the derivation of the conclusions?	1 point	1 point	1 point
Whether the methods used to synthesize the results were appropriate?	1 point	1 point	1 point
Whether to fully assess the possibility of publication bias?	0 points	1 point	1 point
Whether to indicate a conflict of interest?	1 point	1 point	1 point
Total points	9 points	10 points	10 points

**Table 4:** Evidence description and summary.

Intervention measure	Evidence content	Level of evidence	Recommended level
Breast feeding	(1) For neonates, providing breast milk through the nipple or syringe is as effective as using glucose and sucrose [14, 15, 20]	Level 1	Grade A
	(2) For neonates, the smell of breast milk has an analgesic effect [13, 14]	Level 1	Grade B
	(3) Breast feeding should be the first choice for neonatal single operational pain, followed by glucose, sucrose, and other substitutes [20]	Level 1	Grade A
	(4) In full-term neonates, breast feeding has a lower pain response compared with posture, shaking, and mother holding [15]	Level 1	Grade A
	(5) The sweetener of breast feeding is lactose secreted by the mother, which is different from glucose and sucrose [16]	Level 1	Grade A
Sweetener	(6) For neonates, the recommended dose of sucrose for analgesia is 12–120 mg [18]	Level 1	Grade A
	(7) It is recommended that sucrose be taken orally at least 2 min before painful operation [18]	Level 1	Grade A
	(8) Glucose in 20%–30% solution can replace sucrose for analgesic treatment [14]	Level 1	Grade A
	(9) Sweeteners are suitable for infants <3 months [16]	Level 1	Grade A
	(10) Sucrose is advised to be used with caution in preterm infants <32 weeks of pregnancy, unstable condition, and mechanically ventilated neonates [18]	Level 1	Grade A
KMC	(11) KMC can relieve the pain of premature and full-term infants to a certain extent [15]	Level 1	Grade A
	(12) KMC can be performed for neonates who are accustomed to nonbreast feeding [13]	Level 1	Grade A
	(13) Low-birth-weight infants should implement KMC as soon as possible and as long as possible after birth [21]	Level 5	Grade B
	(14) In neonatal KMC, there is no difference in the analgesic effect between mothers and others [19]	Level 1	Grade A
	(15) For neonates, KMC should choose a comfortable position and should be combined with slapping or shaking actions after vaccination [13]	Level 1	Grade A
Sensory stimulation	(16) When all sensory stimulation are used, the analgesic effect is better than that of single oral sucrose [15]	Level 1	Grade A
	(17) The upper limb massage can relieve the pain of neonates [16]	Level 1	Grade A
	(18) During the heel blood collection of premature infants, the pain can be alleviated by playing the same music that the mother heard during pregnancy [15]	Level 2	Grade B
	(19) During neonatal vaccination, the analgesic effect of taste stimulation combined with visual stimulation is better [13]	Level 1	Grade A
Nonnutritive sucking (NNS)	(20) Sweeteners and NNS play a synergistic role in neonatal analgesia [18]	Level 1	Grade A

## Data Availability

The data used and/or analyzed during the current study are available from the corresponding author.
